# A Manual of Diseases of the Nose, Throat, and Ear

**Published:** 1911-01

**Authors:** 


**A Manual of Diseases of the Nose, Throat, and Ear.** By **E. Baldwin Gleason, M.D.,** Clinical Professor of Otology at the Medico-Chirurgical College, Philadelphia. Second edition. 12 mo of 563 pages, profusely illustrated. Philadelphia and London: W. B. Saunders Company. 1910. (Flexible leather, $2.50, net.)

Each revision of Gleason's manual results in a larger, better and more finished book. It is evident from the appearance of the new work that much thought and labor have been expended in its production and by a master in his special field. Among the new or rewritten subjects that have received consideration may be mentioned, new sections on membranous rhinitis, nasal mycosis, septal perforation, Ludwig's angina, Vincent's angina, leprosy of the nose, pharynx, and larynx, the blood in diseases of the upper respiratory tract, intracranial complications of otic disease, and the climatology of diseases of the upper respiratory tract. The section on the tonsils and adenoid structure of the pharynx have been rewritten. The formula at the end of the volume have been thoroughly revised and rearranged, making that part of the book more convenient for reference and more complete.

